# An Indirect Method of Micromagnetic Structure Estimation in Microwires

**DOI:** 10.3390/nano11020274

**Published:** 2021-01-21

**Authors:** Iuliia Alekhina, Valeria Kolesnikova, Vladimir Rodionov, Nikolai Andreev, Larissa Panina, Valeria Rodionova, Nikolai Perov

**Affiliations:** 1 Faculty of Physics, Lomonosov Moscow State University, Leninskie Gory 1-2, 119991 Moscow, Russia; ya.alekhina@physics.msu.ru (I.A.); perov@magn.ru (N.P.); 2 Institute of Physics, Mathematics & IT, Immanuel Kant Baltic Federal University, Gaidara 6, 236041 Kaliningrad, Russia; vakolesnikovag@gmail.com (V.K.); VLRodionov@kantiana.ru (V.R.); andreevn.misa@gmail.com (N.A.); drlpanina@gmail.com (L.P.); 3 Institute of New Materials and Nanotechnology, National University of Science and Technology “MISiS”, Leninsky Avenue 4, 119049 Moscow, Russia

**Keywords:** soft magnetic materials, glass-coated microwires, micromagnetic structure, impedance, magnetic permeability

## Abstract

The tunable magnetic properties of amorphous ferromagnetic glass-coated microwires make them suitable for a wide range of applications. Accurate knowledge of the micromagnetic structure is highly desirable since it affects almost all magnetic properties. To select an appropriate wire-sample for a specific application, a deeper understanding of the magnetization reversal process is required, because it determines the measurable response (such as induced voltage waveform and its spectrum). However, the experimental observation of micromagnetic structure of micro-scale amorphous objects has strict size limitations. In this work we proposed a novel experimental technique for evaluating the microstructural characteristics of glass-coated microwires. The cross-sectional permeability distribution in the sample was obtained from impedance measurements at different frequencies. This distribution enables estimation of the prevailing anisotropy in the local region of the wire cross-section. The results obtained were compared with the findings of magnetostatic measurements and remanent state analysis. The advantages and limitations of the methods were discussed.

## 1. Introduction

Amorphous magnetic materials have been thoroughly studied since the 1970s, mainly for soft magnetic applications at elevated frequencies. Over the past 50 years of research, various methods have been developed for prediction of their properties [1,2] which have provided tremendous technical progress in many areas. Apart from their traditional use in motors due to their low eddy currents losses, other practical applications of amorphous magnetic materials range from magnetic field/stress/temperature sensors to logic, coding and memory systems [3,4,5,6,7,8]. The variety of these applications is due to the specificity of their magnetic and electrical properties (e.g., fast domain wall propagation, giant magnetoimpedance effect) and their sensitivity to external stimuli, such as magnetic field, mechanical load and temperature [9,10,11,12]. In many regards, the ability to precisely control their magnetic structure is the basis for the improvement of related technologies.

The magnetic properties of the sample under investigation depend on its micromagnetic structure, which is influenced by magnetic anisotropy. In amorphous materials, the latter is mostly contributed by magnetoelastic interactions depending on the saturation magnetostriction (both on the magnitude and sign) and the spatial distribution of mechanical stresses arising during production or further processing [2,13,14]. To engineer the magnetoelastic anisotropy in amorphous magnetic materials, different treatment techniques may be applied: annealing, drawing, mechanical processing (see, for example, [15,16,17,18,19,20,21]). The relaxation or redistribution of the mechanical stresses changes the micromagnetic structure, which largely determines the mechanism of magnetization reversal (magnetization rotation, domain wall propagation or magnetization jump). The structural changes reveal themselves in measurable effects such as the shape of hysteresis loops [13], permeability and magnetoimpedance behaviors [22].

Despite the great importance of the micromagnetic structure, the use of methods for its observation has strict limitations. Recently, a novel magnetic tomography method based on X-ray magnetic circular dichroism was proposed [23]. This technique makes it possible to reconstruct the magnetization distribution over the sample and visualize complex micromagnetic structures such as Bloch points, but the linear dimensions of the samples should not exceed several microns. Most of the experimental techniques applicable to bulk magnetic materials allow one to image the micromagnetic structure at the surface [24,25,26]. Some evidence of a particular magnetic structure can be deduced from analysis of the magnetic response from the entire sample or parts of it, for example, from static or dynamic hysteresis loops [15,27,28]. The correct interpretation of such indirect experimental results requires precise control of the experimental conditions as well as a large set of measured patterns typical for a particular type of samples.

Here we consider an alternative indirect method for studying the micromagnetic structure of amorphous wires, which allows one to experimentally inspect the internal domain structure of the sample. The method is based on the impedance measurement at different frequencies.

The impedance of the cylindrical ferromagnetic conductor depends on the permeability with respect to the circular magnetic field (so called, circular permeability) if the skin effect is essential [29]. If this permeability is non-uniformly distributed over the cross-section of the wire, the current density distribution averaged over a specific layer becomes a stepwise function of the averaged permeability. The impedance frequency dependence thus provides information on the permeability averaged over the surface layer which corresponds to the current penetration depth (the layer involved in the current main flow). For this reason, the impedance measurements at several current frequencies allows the reconstruction of the permeability distribution over the cross-section of a wire by fitting the experimental and theoretical data.

In this work, additional techniques were used to deduce the magnetization distribution over the wire cross-section based on magnetization vs. magnetic field behavior. The results were compared to assess the reliability of the approaches and possible errors of the method.

## 2. Materials and Methods

### 2.1. Materials

#### 2.1.1. Amorphous Magnetic Microwires

Amorphous magnetic microwires were chosen as an object of the investigations. Being obtained by rapid solidification from the melt, such materials have a complex micromagnetic structure. Uneven temperature distribution during quenching leads to time-dispersed solidification of various parts of the wire, which causes non-uniform distribution of mechanical stresses. Moreover, for glass-covered microwires the difference in the thermal expansion coefficients of glass and metal induces additional stresses in the wire [30,31]. Combined with magnetostriction, the stress distribution defines the local directions of the easy and hard anisotropy axes and the magnitudes of magnetoelastic anisotropy.

The theoretical consideration of the internal stress distribution in amorphous microwires based on the quenching process draws a two-region image of the metallic part of the wire: the internal core domain with an axially directed magnetization and the shell with a radially or circumferentially directed magnetization, depending on the magnetostriction coefficient sign [32,33]. In addition, a more complex magnetic structure can form in the wire, for example, with a helical magnetization [34,35]. The closure domains arising to minimize the magnetostatic energy should also be taken into account, in particular, for short-length microwires [27,36]. The volume of the inner core domain significantly affects the static and dynamic magnetic properties of microwires: the switching field, the coercivity, the squareness ratio, the range of magnetic fields of single domain wall propagation, and the domain wall mobility. The magnetization distribution also affects the impedance behavior including the change in the shape of impedance vs. magnetic field plots with increasing frequency. Since impedance is a function of the circular permeability, its distribution over the cross-section of the wire has to be taken into account.

#### 2.1.2. Investigated Samples of Amorphous Magnetic Microwires

The amorphous microwires of Co_70_Fe_4_B_13_Si_11_Cr_2_ alloy with near-zero magnetostriction [37,38] produced by Tailor-Ulitovsky technique [39] with different metal cross-section dimensions from 6.4 to 28 µm (6.4, 8, 8.5, 10, 22 and 28 µm) were investigated. The glass shell had the thicknesses of 2.5, 2.8, 2, 2.3, 1, and 3 µm, respectively. The conductivity of amorphous alloy was 8.3 × 10^5^ S. The wire-pieces for measurements were cut by a scalpel.

**Figure 1:**
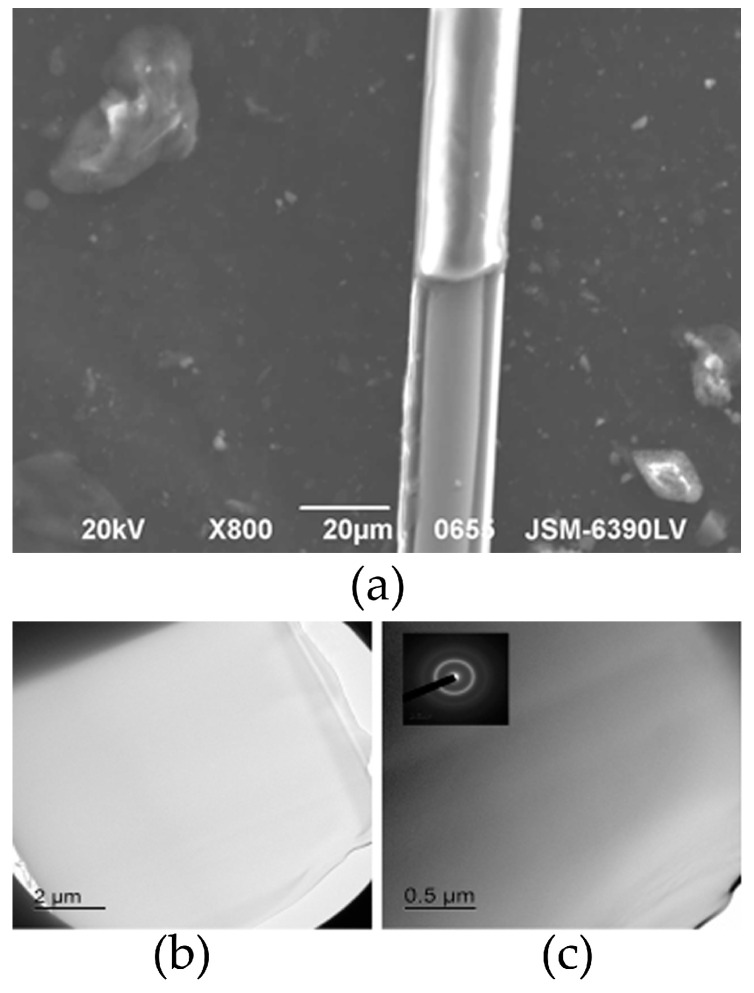
(**a**) SEM and (**b**) TEM images of Co_70_Fe_4_B_13_Si_11_Cr_2_ microwire. (**c**) SAED from the microwire and subsequent dark-field image obtained in the first diffraction ring. The samples demonstrate an amorphous structure.

Because the wire magnetic properties are extremely sensitive to the cross-section size and the phase state (e.g., existence of nanocrystalline clusters which depends on the initial technical parameters [40,41]), we carefully examined the microwire samples using a scanning electron microscope (JSM-6390LV, Jeol, Tokyo, Japan) to determine the relevant dimensions and the transmission electron microscope (JEM-2100 Jeol, Tokyo, Japan, with an accelerating voltage of 200 kV) to detect any presence of nanocrystalline areas. The SEM images are presented in Figure 1a and the TEM micrographs of a thicker wire sample are depicted in Figure 1b,c. A selected area diffraction (SAED) represents an amorphous halo. The dark-field observation in the first diffraction ring also confirms the amorphous structure.

### 2.2. Methods

For the microstructure investigations, two basic approaches were used. The first is based on the analysis of the impedance characteristics at different frequencies to reproduce the permeability distribution, and the second utilizes the analysis of hysteresis loops: remanent magnetization and re-magnetization mechanisms.

#### 2.2.1. Impedance of the Wires and Permeability Calculation

##### Theoretical Background

For moderate frequencies (not very strong skin effect), the impedance of a magnetic wire with a spatially independent permeability tensor is of the form [29]:

(1) $$Z=R_{DC}\frac{ka}{2}\frac{J_{0}\left(ka\right)}{J_{1}\left(ka\right)}$$

$$k=\;\frac{1-i}{\delta },\;\delta =\;\frac{1}{\sqrt{\pi f\sigma \mu_{0}\mu_{\varphi }}},$$

where $R_{DC}$ is the *DC* resistance, $J_{0}$ and $J_{1}$ are the Bessel functions of zero and first orders, respectively, δ is the skin depth, f is the current frequency, σ is the wire conductivity, $\mu_{0}$ is the vacuum permeability, and $\mu_{\varphi }$ is the circular permeability, a is the wire radius.

This expression explains the typical shapes of the giant magnetoimpedance (GMI) curves. The magnetic field dependence of the circular permeability, included in Equation (1) as the function argument, is determined by the magnetic anisotropy. A bell-shaped curve of GMI is associated with an axial anisotropy, whilst a circular anisotropy results in GMI plots with two symmetrical peaks appearing at the external field nearly equal to the anisotropy field [22]. In some cases, CoFe-based microwires show asymmetry in the GMI plots. Such behavior is explained in terms of the combination of a helical magnetic anisotropy and a circular field produced by the bias current during the measurements [42,43].

Equation (1) for the impedance is obtained considering a linear relationship between AC magnetization and magnetic field under the assumption of a spatially independent permeability tensor. In general, the permeability parameter $\mu_{\varphi }$ is composed of the components of the permeability tensor. In real samples the permeability may be distributed over the volume, and its dependence on the magnetic field (which is also non-uniformly distributed over the wire cross-section) have to be taken into account. It is assumed, that for a smooth radial dependence of permeability the same expression works if the parameter $\mu_{\varphi }$ is replaced by an average permeability of the layer related to the skin depth. For the detailed reconstruction of the permeability distribution in complex cases of the dependence μ(r, H), the calculation method has to be sufficiently modified. A simplified approach makes it possible to deduce some tendencies of the permeability spatial distribution.

In the first approximation, assuming a complex-valued permeability $\mu_{\varphi }=\;\mu_{\varphi }^{\prime }-i{\mu_{\varphi }}^{\prime \prime }$, the real and imaginary parts can be reconstructed by solving the inverse problem. The minimum position of the function ∆Z:

(2) $$\left|∆Z\right|=\left|Z_{exp}-\;Z_{th}\right|$$

where $Z_{exp}\;$ is the measured impedance and $Z_{th}$ is the theoretical value determined by Equation (1), on {$\mu_{\varphi }^{\prime },\;{\mu_{\varphi }}^{\prime \prime }$} surface corresponds to the permeability value averaged over the wire layer, where the bulk of the current flows. We assume that 70% of the current flow is the main contributor.

**Figure 2:**
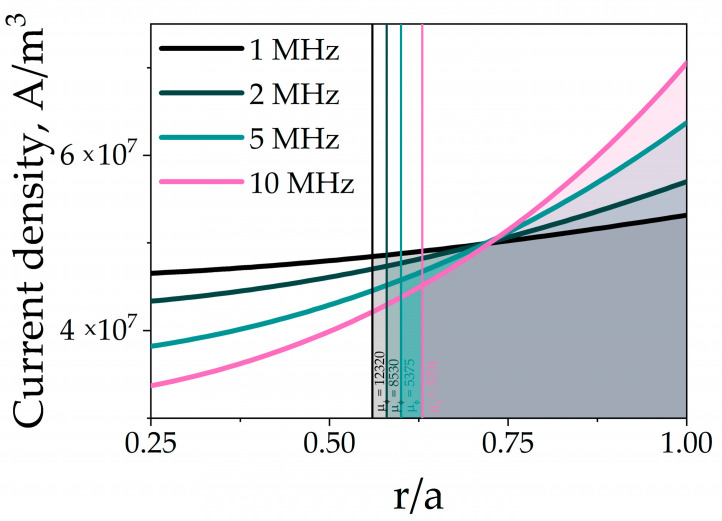
Radial dependence of the current density at frequencies of 1, 2, 5 and 10 MHz. The vertical lines bound the layers through which 70% of the total current flows (shaded areas). The average permeability values for each case are marked with the corresponding color (these values were found by experimental data fitting procedure). For a frequency 1 MHz, the permeability $\mu_{\varphi }$ is of the order of $10^{4}$, which is typical for low-magnetostriction wires.

The current density in the wire depends on the radius due to the skin-effect. The current integrated over the surface layer of thickness h= a−r equals:

(3) $$I_{h}=\;I_{0}\left(1-\frac{r}{a}\frac{J_{1}\left(kr\right)}{J_{1}\left(ka\right)}\right),\;\;$$

where $I_{0}$ is the total current passing through the wire. Using Equation (3), the current penetration depth h_70%_ where 70% of the current flow is concentrated, can be estimated. As an example, for a sample with a diameter of 8 μm the current density distribution at various frequencies from 1 to 10 MHz is shown in Figure 2, where the penetration depth and average permeability are also indicated.

The AC current passing through the wire produces an AC circular magnetic field, which causes the AC magnetization. In the presence of a DC magnetic field, the AC magnetization process occurs by the both mechanisms: magnetic moment rotation and domain wall movement. With increasing frequency towards MHz range the main contribution comes from the magnetization rotation as the domain walls are damped [44,45].

**Figure 3:**
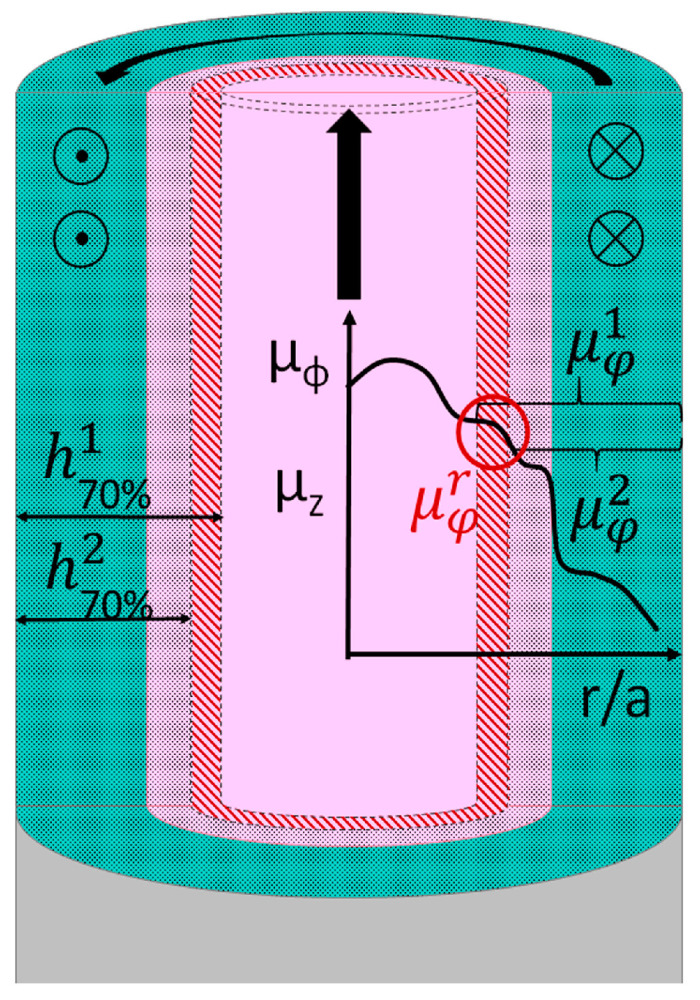
Schematic of the permeability distribution calculation based on averaging the permeabilities over the penetration depths. The values of the average permeability $\mu_{\varphi }^{1}$ and $\mu_{\varphi }^{2}$ correspond to the experimental impedance measured at two frequencies $f(f_{1}\;<\;f_{2})$. The permeability values are used to estimate the corresponding penetration depths $h_{70\%}^{1}$ and $h_{70\%}^{2}$. From average values, the permeability $\mu_{\varphi }^{r}\;$ of the differential layer (red) can be interpolated.

Varying the frequency, the changes in h_70%_ and corresponding $\mu_{\varphi }$ can be obtained. As measured $\mu_{\varphi }$ is an average value of permeability over the layer with thickness h_70%_, knowing the array of $\mu_{\varphi }$ corresponding to different h_70%_, one can reconstruct the radial dependence of permeability $\mu_{\varphi }^{r}$, as depicted in Figure 3.

If the current penetration depth falls into the axially magnetized region, the average permeability associated with the magnetization rotation increases due to the perpendicular configuration between the DC magnetization and AC magnetic field. We assume that this enhanced permeability corresponds to the axial anisotropy, which also affects the magnetization reversal process and can be revealed in hysteresis experiments. Consequently, analyzing the radial dependence of permeability and identifying abrupt changes in $\mu_{\varphi }$, it is possible to trace the regions in which the prevailing type of anisotropy changes.

For the permeability calculation the following procedure was used:

(i) using the experimental data on the wire impedance, the real and imaginary parts of permeability were determined from Equations (1) and (2);
(ii) knowing the permeability value, the corresponding current penetration depth at every frequency was obtained from Equation (3);
(iii) for each subsequent pair of permeability and penetration depth values the average permeability for the differential layer (red layer in Figure 3) was obtained. The values obtained were presented in the form of a histogram, where the pillar height and width represent the local permeability and the thickness of the differential layer, respectively.

#### 2.2.2. Magnetostatic Measurements

There are several indirect experimental methods for obtaining data for estimating the volume of the axially magnetized core of the microwire based on magnetostatic measurements. Local hysteresis loops along the microwire axis can be obtained by measuring the magnetic flux in short movable pick-up coils. This also allows the magnetization profile to be measured in order to assess the contribution of closure domains [15,27,46,47].

When using integrated fluxmetric methods for studying magnetic properties, the closure domains do not contribute to the measured hysteresis loop. The estimation of core domain volume may be more accurate comparing to that obtained using vibrating sample magnetometry methods (VSM). However, the AC fields are used in the fluxmetric methods, which causes the magnetic parameters to vary with a frequency and an instant value of the excitation magnetic field [28].

In the case of using the VSM methods, the closure domains contribute to the measured hysteresis loops. Moreover, in a Vector VSM setup, the two pairs of pick-up coils are located at some fixed distance from each other and the sample is placed between them. This means that different parts over the length of the elongated specimen have different contributions to the magnetic moment projections and the final estimate of the volume of the axially magnetized core is either too large or too small.

**Figure 4:**
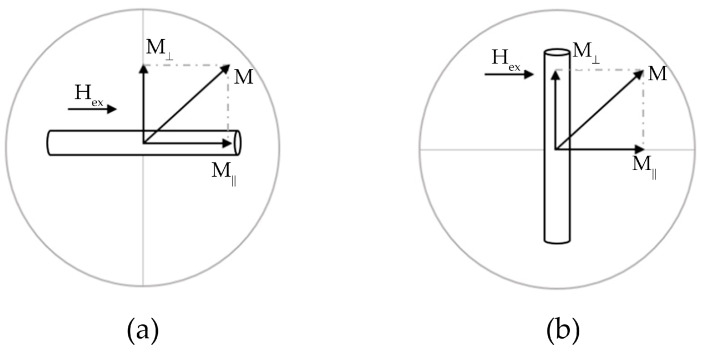
Schematic diagram showing the principal directions of the measured magnetic moment with respect to the applied magnetic field H_ex_: (**a**) component of the wire magnetic moment along the field H_ex_ as a function of H_ex_, (**b**) cross-magnetic moment vs. H_ex._

In this work, VSM with a modified pick-up coil configuration was used for magnetostatic measurements. We used double half-ring coils having a diameter of 2 cm and $10^{4}$ turns of copper wire with a thickness of 30 µm, as schematically shown in Figure 4 by grey line. The proposed pick-up coil arrangement offers a possibility of measuring different projections of the magnetic moment keeping the same value of the magnetic flux from the sample [48].

The measurements of (a) conventional hysteresis loops, that is, the component of the wire magnetic moment along the magnetizing field H_ex_ as a function of H_ex_ and (b) perpendicular hysteresis loops, that is, the component of the wire magnetic moment perpendicular to H_ex_ vs. H_ex_ (named as cross-magnetic moment) were carried out. The measurement geometry s for both cases is demonstrated in Figure 4: the axial magnetic moment was measured with respect to the magnetic field applied along the wire (a) and perpendicular to the wire axis (b).

**Figure 5:**
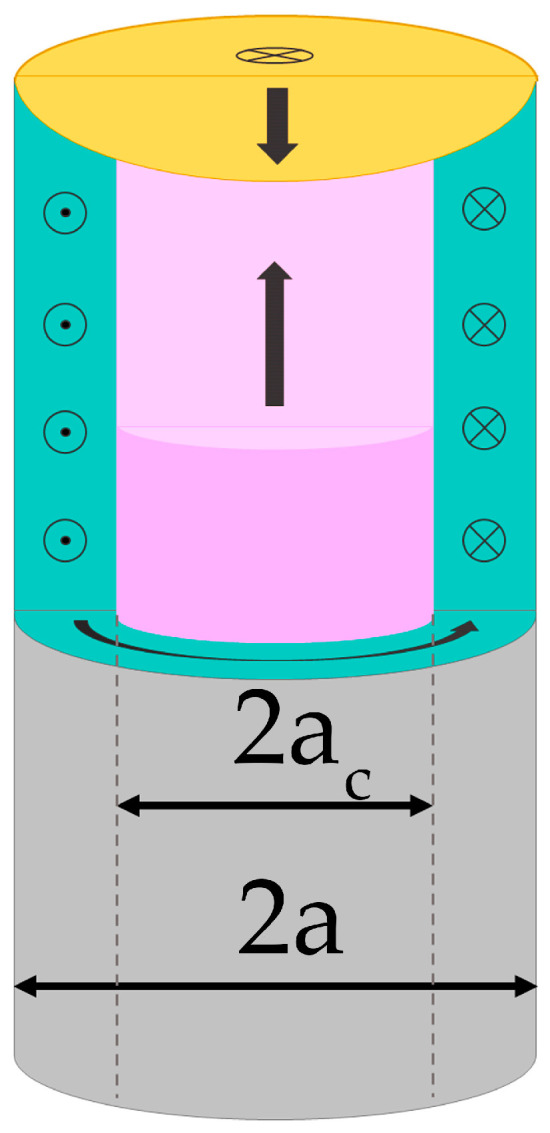
Schematic of the micromagnetic structure of the metallic part with a diameter 2a of a magnetic microwire: axially magnetized core of a diameter 2a_c_ (pink) and circularly magnetized shell (green). Closure domains are presented by the yellow region.

Radius estimates of the core domain can be made using two algorithms. The first one is based on the analysis of the remanent magnetization measured along the external field H_ex_ as in (a) case. For this configuration, the remanent magnetization M_r_ corresponds to the core domain magnetization (with the contribution of closure domains) and the saturation magnetization is a characteristic of a uniformly magnetized wire. Figure 5 shows a typical micromagnetic structure of the metallic part of a glass-coated microwire, from which it can be seen that the squareness ratio of the hysteresis loop $\frac{M_{R}}{M_{S}}$ defines the volume fraction V_C_ of the axially magnetized core with respect to the entire sample volume V_S_. Thus, the radius of the axially magnetized core can be estimated.

The second method uses the results of hysteresis loops measurements in both configurations (a) and (b). This requires the comparison of the maximum values of the magnetic moment components measured in parallel (a) and perpendicular (b) orientations. The maximum magnetic moment in case (a) corresponds to M_S_. The maximum value of the cross-magnetic moment, M_C_, is the magnetic moment of the axially magnetized core only originated from the axial anisotropy. This excludes the closure domains contribution. Similar to the squareness coefficient, their ratio shows the volume fraction of the core:

(4) $$\frac{M_{S}}{M_{C}}=\frac{V_{S}}{V_{C}}\;$$

Thus, we can estimate the location of the domain wall between the axially magnetized core and the circumferentially magnetized shell inside the microwire:

(5) $$\frac{V_{s}}{V_{c}}=\frac{a^{2}}{a_{c}^{2}}$$

Using Equation (5), the radius of the axially magnetized core can be expressed by:

(6) $$a_{c}=a\sqrt{\frac{V_{c}}{V_{s}}}$$

#### 2.2.3. Experimental Equipment

The measurements of the impedance $Z_{exp}$ were carried out using a HP4395A network/spectrum/analyzer (Hewlett-Packard, Palo Alto, CA, USA) which was calibrated to get the data of high resolution in MHz frequency range. The measurements were carried out when an alternating current with an amplitude of 2.5 mA kept fixed by the hardware and a frequency of *f* = 0.5–10 MHz was passing through the sample. The length of the microwire sample was 8 mm.

The calculations using the procedure described in Section 2.2.1 were carried out with the use of MatLab R2015b (MathWorks, Santa Clara, CA, USA) software.

The measurements of magnetic moment were carried out using self-assembled VSM with double-split measuring coils [48]. The magnetic moment resolution was 10^−3^ Am^2^. The range of the applied magnetic field was ±800 kA/m. The field increment in the low-field range was 2.4 A/m. The measured magnetic moments were given in arbitrary units, which correlated with the induced voltage in the pick-up coils. The length of microwire samples was 1.5 cm which is sufficient to maintain the original micromagnetic structure for microwires with mentioned diameters and compositions [49].

## 3. Results and Discussion

### 3.1. Conventional Hysteresis Loops

**Figure 6:**
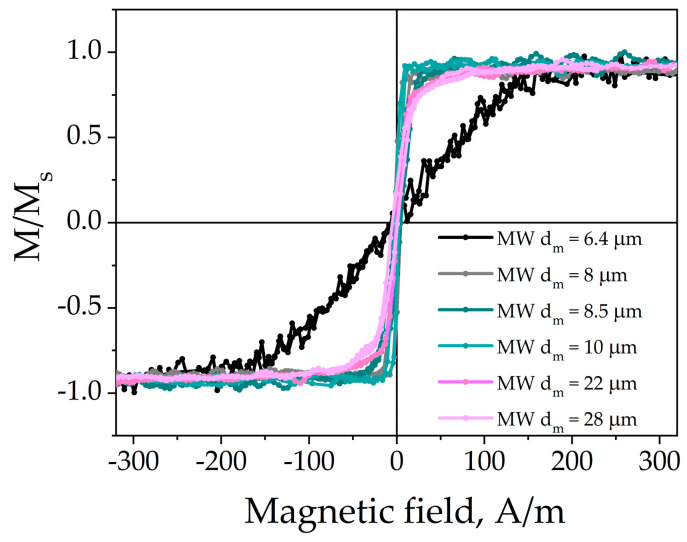
Normalized hysteresis loops of the Co_70_Fe_4_B_13_Si_11_Cr_2_ amorphous glass-coated microwires with different diameters. The shape of hysteresis loops evolves depending on the diameter of the metal core.

The shape of the hysteresis loops is a good indicator of the magnetization mechanism. Figure 6 compares the hysteresis loops of CoFe-based wires with different metallic core diameters measured in (a) geometry (see Figure 4). They all have low coercivity (lower than 5 A/m) typical of good soft magnetic materials. The saturation field does not exceed 40 A/m. The squareness ratio is from 0.02 to 0.45. The parameters are listed in Table 1. The radii of the axially magnetized core were calculated for each sample from the squareness ratio and varied from 0.05 to 0.67 of the total core radius.

### 3.2. Field Dependence of the Cross-Magnetic Moment

**Figure 7:**
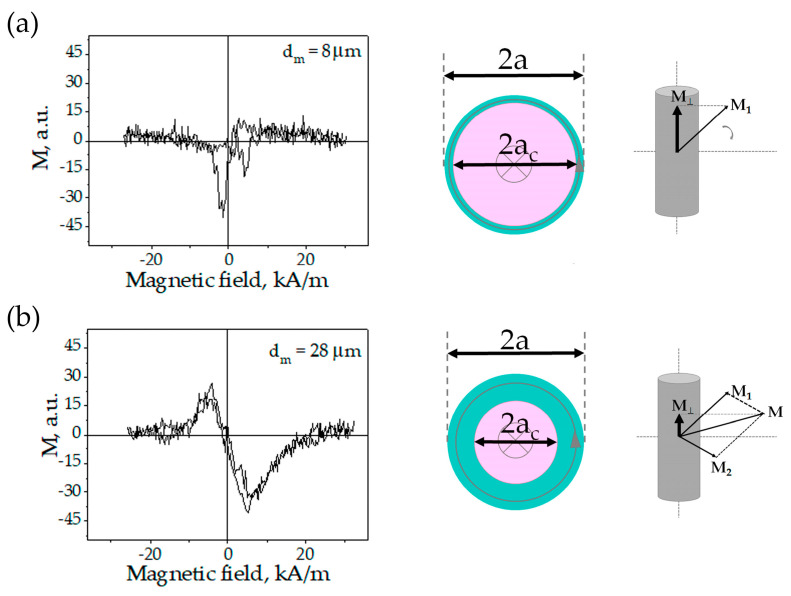
Axial magnetic moment vs. magnetic field applied perpendicular to the wire axis for $d_{m}=8\;\mu m$ in (**a**) and 28 μm in (**b**).

Figure 7 shows the plots of the axial magnetic moment vs. the field applied perpendicular to the wire axis (cross magnetic configuration). In the saturation field this component tends to zero since the wire is magnetized in perpendicular direction. The magnetization mechanisms are illustrated in Figure 7 (right column) where $M_{1}$ and $M_{2}$ are the magnetic moment of the core (with the axial anisotropy) and the periphery, respectively.

Figure 7a clearly demonstrates the existence of the domain processes in the region of low magnetic fields resulting in a sharp peak of the cross-magnetic moment. The axial orientation of the magnetization is assisted by the perpendicular field owing to the corresponding components of the internal stress. Figure 7b is consistent with the rotational mechanism of the magnetization. The values of the magnetic moments in Figure 7a,b should not be compared as they are obtained for microwires with different diameters. For further analysis only relative values are of interest. Analyzing the relative data for the two types of magnetization curves, it will be possible to draw conclusions about the distribution of the easy anisotropy axes (either axial or circumferential) within the microwire cross-section.

### 3.3. Remanent Magnetization State Analysis

Using the results of the two types of measurements (conventional hysteresis loops and cross-magnetization loops), we estimated the volume of the axially magnetized core for all samples. The results are summarized in Table 2. The differences in estimations are clearly seen. The reason is the difference in the magnetization processes of the wire when a magnetic field is applied parallel or perpendicular to the wire axis.

Based on the squareness coefficient, we could assume that the microwire with a diameter $d_{m}\;$= 28 µm has quite a large axial domain volume comparable with that of thinner wires (0.6 a). On the other hand, the axial magnetic moment tends to zero when the perpendicular field decreases to zero. This indicates that the magnetic moment of the remanent state at conventional measurements can be decreased due to the contribution of closure domains. It means, that for samples with large closure domains the core domain radius cannot be accurately found on the basis of the squareness coefficient. The cross-magnetic moment measurements provide more accurate information about the remagnetization mechanism and prevailing anisotropy allowing the magnetization evaluations without the closure domain contribution.

### 3.4. Magnetoimpedance

**Figure 8:**
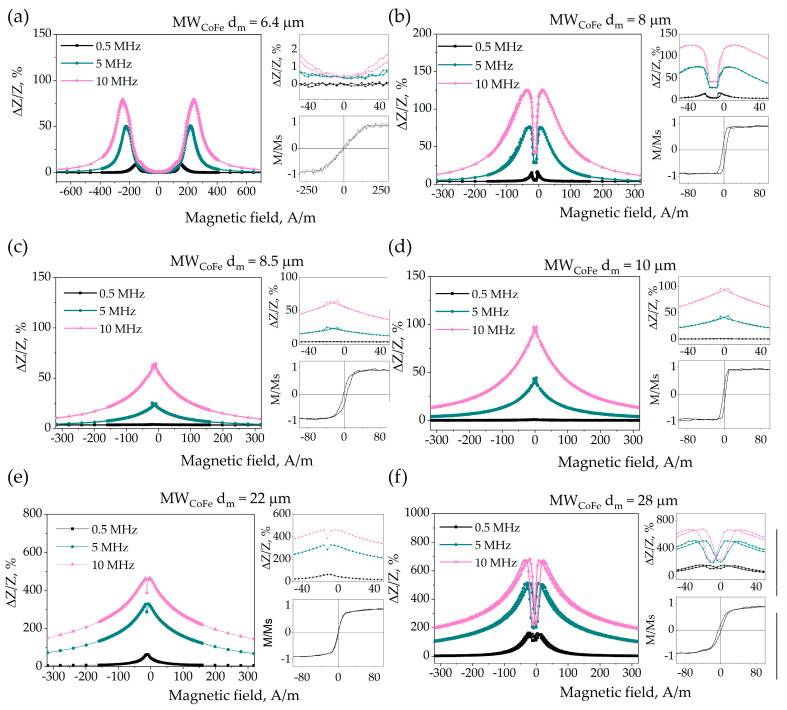
GMI field dependences at different frequencies for microwires with different metallic core diameters from 6.4 to 28 μm (**a**–**f**). Enlarged near-zero field region and hysteresis loops for the samples are included. Evolution of GMI curve shape from double peak (**a**,**b**) to single-peak (**c**,**d**) and back to double-peak (**e**,**f**) with increasing metal core diameter is observed.

GMI dependences for all the samples were analyzed in order to get the information about the prevailing anisotropy type and for further establishments of model working frames (Figure 8). A two-peak GMI curve is inherent of wires with a circular anisotropy. When axial or radial anisotropy prevails, a single-peak dependence is usually observed [22].

For the wires under investigation the shape of the GMI curve evolves with the increase in the metallic core diameter. For the thinnest sample the two-peak plots were observed. When the diameter of the wire increases, the field of the GMI maximum shifts to lower values, and for samples with d_m_ = 8.5 and 10 µm the dependence with a single peak was observed. With further d_m_ increase, a single peak splits again into two peaks. This demonstrates a non-monotonic change in the domain structure with an increase in the wire diameter, which can be explained by the difference in the stress distribution and the dependence of magnetostriction on stresses.

It was reported, that for amorphous alloys the magnetostriction coefficient depends on the magnitude of the mechanical stresses. For materials with nearly zero magnetostriction it can change the sign under the mechanical influence [50,51,52,53]. As the internal stresses in the wire depend on the geometrical and manufacture parameters, the wires of the same compositions but prepared at different conditions may have opposite signs of the magnetostriction coefficient and, thus, different types of the domain structure [50,54,55].

The GMI curves for microwires with d_m_ = 6.4, 8, 22 and 28 µm have two peaks, that is, they have a predominant circular anisotropy. For the thinnest sample (d_m_ = 6.4 µm) there is a wide plateau around zero field, which is associated with a strong circular anisotropy field [56]. The shape of the hysteresis loop (Figure 8a) and the absence of cross-magnetic moment indicate that the wire has no core in the domain structure. The GMI curve for the sample with d_m_ = 8 µm also demonstrates two peaks with narrow plateau. Taking into account the shape of cross-magnetic moment curve, it is supposed, that the regions of axial and circular magnetization coexist in this wire. For the samples with d_m_ = 22 and 28 µm both the cross-magnetic moment loops and GMI curves allow us to conclude, that circular magnetization predominates.

For the samples with d_m_ = 8.5 and 10 µm a single-peak GMI curve is observed. The comparison of GMI with the results of magnetostatic measurements for these samples showed, that there was a large fraction with an axial magnetization in the wires. Considering the GMI curves and possible stress-dependence of the magnetostriction it is assumed, that internal stresses induced a change in the magnetostriction sign, and the radial magnetization distribution prevails in the shell of these samples.

### 3.5. Radial Distribution of Magnetic Permeability

**Figure 9:**
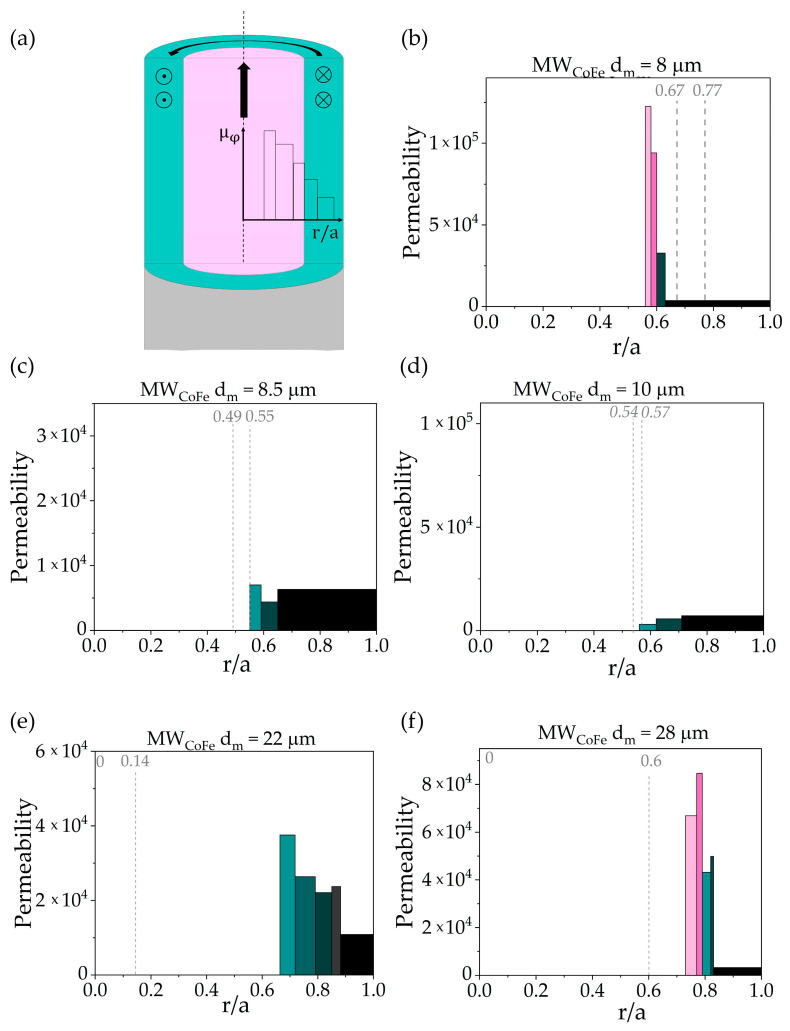
Radial distribution of the permeability of CoFe-based microwires with different diameters. (**a**) Schematic representation of the permeability histogram construction reflecting radial permeability distribution, (**b**–**f**) permeability distribution histograms for the samples with different metal core diameters. The height of the bar is the local permeability value and the width is the thickness of the corresponding layer. Color is used to contrast the permeabilities of different layers. The permeability calculation was done for zero DC magnetic field.

Figure 9 demonstrates the radial distribution of circular permeability for all samples. Since the impedance of the wires was measured at MHz frequencies, the main contribution to the permeability comes from the magnetization rotation, while the motion of domain walls is suppressed by eddy currents. This is confirmed by previous studies of the frequency behavior of permeability in microwires of similar composition [45]. However, at zero DC field and circular anisotropy, the AC circular field does not cause magnetization rotation and only the displacements of circular domain walls affect the permeability. The current density distribution at various frequencies from 1 to 10 MHz and corresponding current penetration depths and average permeability values for the sample with 8 μm diameter are presented above in Figure 2.

The results obtained demonstrate, that for the thinnest sample with a diameter of 6.4 µm the permeability value cannot be correctly calculated, as the rapid changes in impedance with frequency are consistent with a negative average permeability of the wire. This dependence can arise due to large heterogeneity of the permeability as well as the contribution of regions with axial anisotropy. As the magnitude of stresses affects the local anisotropy [32,33], the permeability is also sensitive to the stress distribution. In the case of large internal stresses, such as in thin wires [51], the radial dependence of permeability can be very sharp, and averaging provides large errors. In this case, the permeability spatial distribution and field dependences have to be taken into account in Maxwell’s equations, and the first approximation is not applicable. For the samples with a diameter of 8.5 and 10 µm, the calculated permeabilities have relatively low values at zero DC magnetic field, and the radial dependence is not pronounced. The GMI curves measured for these samples correspond to the case of a radial magnetization distribution in the shell. The shapes of the hysteresis loops (which are presented in Figure 7 suggest, that radial anisotropy is formed in these samples, as the saturation field does not exceed 40 A/m. When the current passes through the sample, the processes of non-uniform magnetization rotation occurring in a radially magnetized shell may enhance losses and, thus, the imaginary part of the permeability. Since such processes are not taken into account in our model, the real values of permeability have to be much higher, than the calculated ones. It means, that such a simplified model cannot be used for microwires with a radially magnetized shell.

The permeability distribution calculated for the sample with a diameter of 8 µm shows a decreasing trend with increasing r. The sharp decrease in the permeability radial dependence occurs at a distance of around 0.60 of radius. The diameters of an axially magnetized core obtained from the squareness ratio and cross-magnetic moment were 0.67 a and 0.77 a, respectively. Consequently, an abrupt change in the circular permeability can be associated with a change of prevailing anisotropy type from axial to circular at the boundary between the core and the shell. For a more accurate determination of the core radius, fine sampling of the layers is required.

For a microwire with a diameter of 22 µm the circular permeability decreases monotonically with increasing r without sharp jumps. The absence of permeability jumps indicates, that the wire region under investigation have rather uniform micromagnetic structure. Presumably, all the averaging layers are located in the shell region. This result is also consistent with the core radii, obtained using the cross-magnetic moment or squareness ratio techniques.

For a sample with a metal diameter of 28 µm the permeability distribution has a sharp decrease at 0.82 of the wire radius. As the cross-magnetic moment signal indicated the absence of the core domain, and the squareness coefficient gave the value of ~0.14 a for the core domain radius, the results may be explained by the formation of the surface layer with much higher circular anisotropy.

A brief description of the results is presented in Table 3. Detailed table with the experimental results for all investigated samples is presented in the Supplementary Material.

All the described techniques for estimating the core radius showed the consistent results in cases of the circular anisotropy in microwires. Nevertheless, there are several subtleties in the applicability and accuracy of the methods, which have to be considered.

The impedance measurement in a wide frequency range with subsequent calculation of the magnetic permeability distribution allow one to trace the changes of the magnetization distribution caused the presence of interfaces between regions with different type of anisotropy or defects. This method allows one to reconstruct the data on the microstructure of a sample based on the experimental results, which makes it promising for microtomography of samples with a complex domain structure. The performed analysis shows, that this model provides reasonable results in the case of circular anisotropy in the shell if the current amplitude is low and does not cause the non-linear AC magnetization. In cases of significant permeability variations with radial coordinate or radial/axial anisotropy additional magnetization mechanisms have to be taken into account.

## 4. Conclusions

In this paper we have presented a novel way to estimate the micromagnetic structure in microwires, in particular, to determine the permeability distribution in the cross-section of the wire. The method is based on the dependence of the impedance of a cylindrical conductor on its circular permeability: from the frequency dependence of the impedance the permeability value for each current penetration depth was calculated in the first approximation of non-constant permeability effect on the impedance. Analysis of the radial distribution of permeability made it possible to determine the position of boundary separating the core and shell of the wire with different anisotropy. The results were compared with those obtained by two magnetostatic methods involving different components of the microwire magnetic moment. For this, the conventional hysteresis loops and the magnetic field dependence of the cross-magnetic moment were measured. For the sample with metallic core diameter of 8 µm and circular magnetization in the shell region the calculated core radius corresponded to that obtained using two magnetostatic methods. Similar results were obtained for the thicker sample with 22 µm diameter. For the sample with the largest diameter 28 µm the permeability have strongly non-uniform distribution over the cross-section. For the rest of the samples the sharp permeability variations with radial coordinate and radial anisotropy limited the method applicability. For such samples, simplified approach does not provide information about the permeability value, additional magnetization mechanisms have to be taken into account.

It is concluded that the proposed method can be used for microwires with circular anisotropy and with a smooth change in permeability over the cross-section. The results obtained correlate with the conventional method based on the squareness ratio estimation but at the same time give additional information about the microstructure or local defects, which has great potential for microtomography and defectoscopy.

## Figures and Tables

**Table 1:** Magnetic parameters of the Co_70_Fe_4_B_13_Si_11_Cr_2_ amorphous microwires.

Metallic Core Diameter, d_m_ [μm]	Coercivity, H_C_ [Oe]	Remanence to Saturation Ratio, M_R_/M_S_
6.4	0.05	0.05
8	0.05	0.45
8.5	0.05	0.24
10	0.03	0.33
22	0.04	0.35
28	0.003	0.02

d_m_ is the diameter of the metallic core; *H_C_* is the coercive field; *M_R_/M_S_* is the squareness ratio, *M_R_* is the remanent magnetization, *M_S_* is the saturation magnetization.

**Table 2:** Estimated radius of axially magnetized core by two methods.

Metallic Core Diameter, d_m_ [μm]	Core Domain Radius, a_c_/a	Core Domain Radius, a_c_ */a
6.4	~0	0.22
8	0.77	0.67
8.5	0.55	0.49
10	0.54	0.57
22	~0	0.60
28	~0	0.14

d_m_ is the diameter of the metallic core; a_c_ is the radius of the axially magnetized core of the microwire metallic part; a_c_ * is the radius of the axially magnetized core of the microwire metallic part calculated on the basis of squareness ratio; a is the radius of the metallic part.

**Table 3:** The results of indirect method of micromagnetic structure evaluation.

d_m_ [μm]	Structure	Method Applicability
6.4	Circular without core, sharp permeability radial dependence	Not applicable
8	Circular	Applicable, core radius calculated—0.60 R
8.5	Axial	Not applicable
10	Axial	Not applicable
22	Circular	Applicable, measurements in the shell
28	Circular	Applicable, non-uniform permeability

## Data Availability

The data presented in this study are available in the article and Supplementary Material.

## Supplementary Materials

The following are available online at https://www.mdpi.com/2079-4991/11/2/274/s1, Table S1: Experimental data on the samples under investigation: magnetization curves, cross-magnetic moment dependences, GMI curves and the permeability radial dependence.
