# Supplementary material for: An Indirect Method of Micromagnetic Structure Estimation in Microwires
Source: Nanomaterials (Basel). 2021 Jan 21;11(2):274. doi: 10.3390/nano11020274 (PMC7911699; doi:10.3390/nano11020274)
Supplement: Supplementary file 1 [file nanomaterials-11-00274-s001.pdf]

nanomaterials

MDPI

Table S1: Experimental data on the samples under investigation: magnetization curves, cross-magnetic moment dependences, GMI curves and the permeability radial dependence.

Sample	Magnetization curve	Cross-magnetic moment	GMI curves for f = 0.5, 5 и 10 MHz	Radial permeability distribution
d_m = 6.4 μm	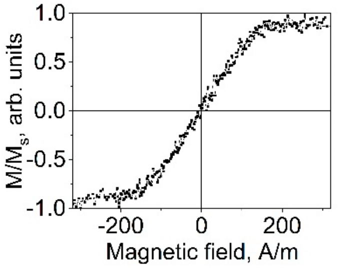	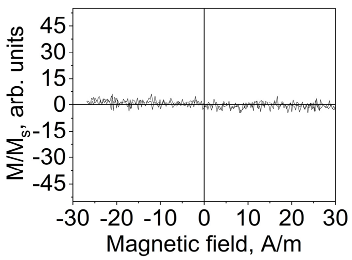	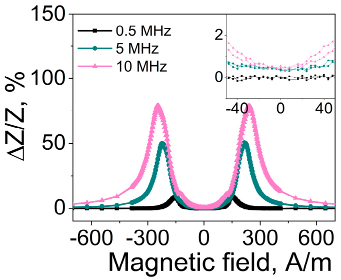	Cannot be calculated
d_m = 8 μm	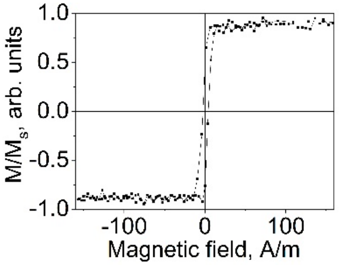	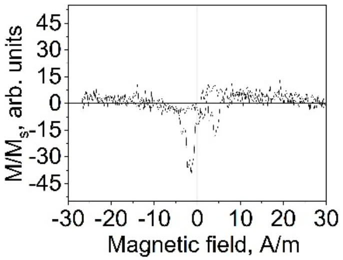	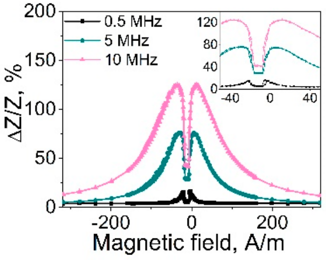	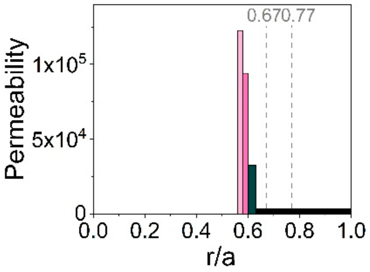
d_m = 8.5 μm	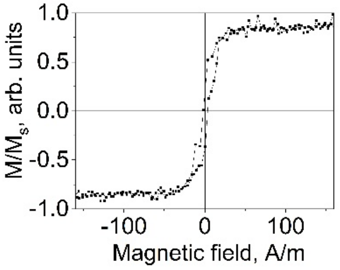	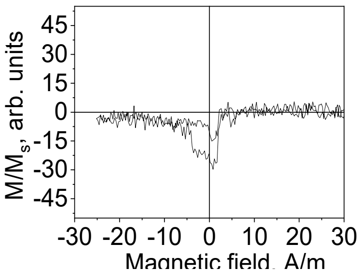	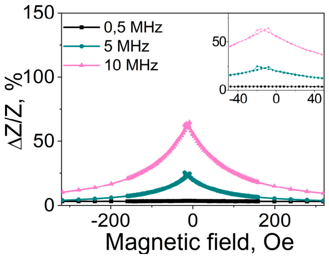	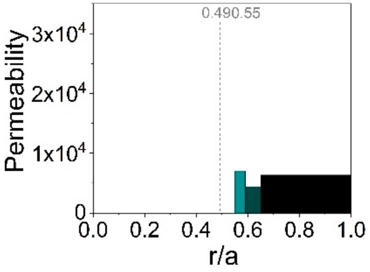

<table>
<tr><th>Sample</th><th>Magnetization curve</th><th>Cross-magnetic moment</th><th>GMI curves for f = 0.5, 5 и 10 MHz</th><th>Radial permeability distribution</th></tr>
<tr><td>d_m = 10 μm</td><td>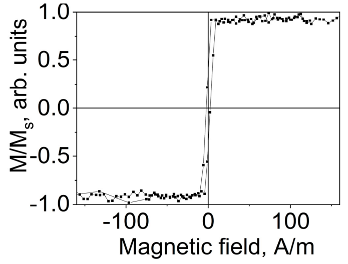</td><td>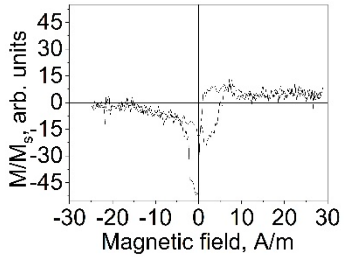</td><td>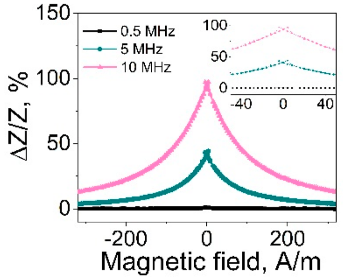</td><td>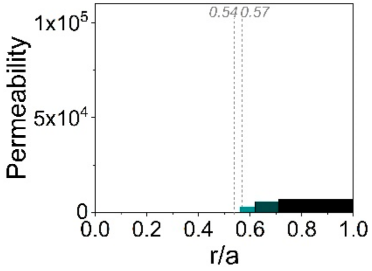</td></tr>
<tr><td>d_m = 22 μm</td><td>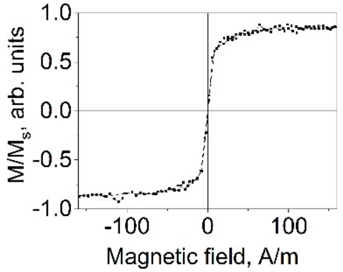</td><td>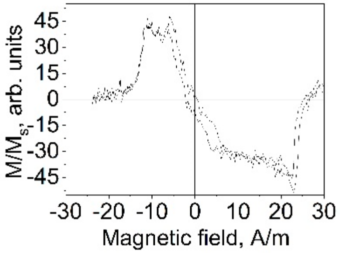</td><td>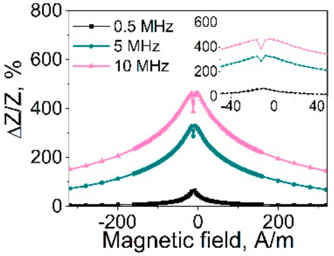</td><td>5
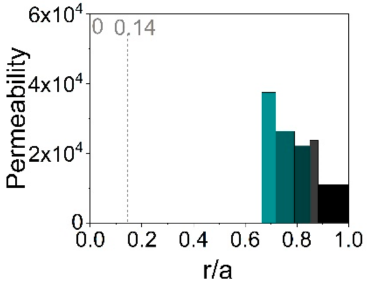</td></tr>
<tr><td>d_m = 28 μm</td><td>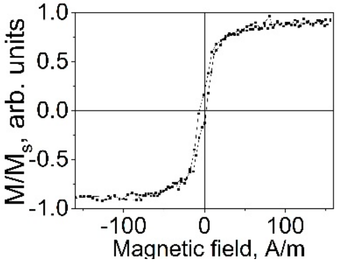</td><td>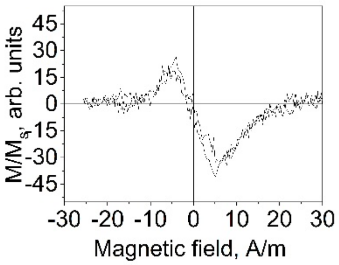</td><td>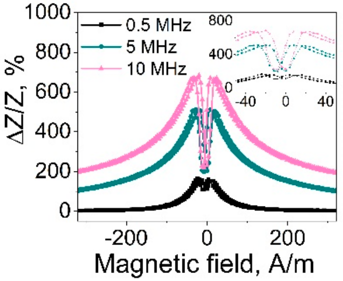</td><td>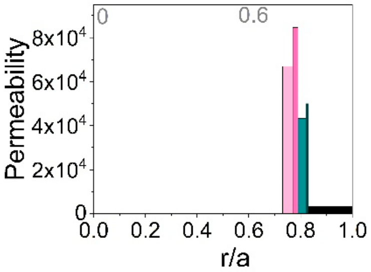</td></tr>
</table>
